# Characteristics and survival for HIV-associated multicentric Castleman disease in Malawi

**DOI:** 10.7448/IAS.18.1.20122

**Published:** 2015-08-03

**Authors:** Satish Gopal, N George Liomba, Nathan D Montgomery, Agnes Moses, Bongani Kaimila, Richard Nyasosela, Maria Chikasema, Bal M Dhungel, Coxcilly Kampani, Marcia K Sanders, Robert Krysiak, Dirk P Dittmer, Yuri Fedoriw

**Affiliations:** 1 UNC Project-Malawi, Lilongwe, Malawi; 2 Program in Global Oncology, Lineberger Comprehensive Cancer Center, Chapel Hill, NC, USA; 3 Department of Medicine, Institute for Global Health and Infectious Diseases, University of North Carolina at Chapel Hill, Chapel Hill, NC, USA; 4 Department of Medicine, University of Malawi College of Medicine, Blantyre, Malawi; 5 Division of Hematopathology, Department of Pathology and Laboratory Medicine, University of North Carolina at Chapel Hill, Chapel Hill, NC, USA; 6 Kamuzu Central Hospital, Lilongwe, Malawi; 7 United Nations Development Program, Lilongwe, Malawi; 8 Department of Microbiology and Immunology, University of North Carolina at Chapel Hill, Chapel Hill, NC, USA

**Keywords:** HIV, multicentric Castleman disease, Kaposi sarcoma, Kaposi sarcoma-associated herpesvirus, non-Hodgkin lymphoma, sub-Saharan Africa

## Abstract

**Introduction:**

Clinical reports of multicentric Castleman disease (MCD) from sub-Saharan Africa (SSA) are scarce despite high prevalence of HIV and Kaposi sarcoma-associated herpesvirus (KSHV). Our objective is to describe characteristics and survival for HIV-associated MCD patients in Malawi. To our knowledge, this is the first HIV-associated MCD case series from the region.

**Methods:**

We describe HIV-positive patients with MCD in Lilongwe, and compare them to HIV-associated lymph node Kaposi sarcoma (KS) and non-Hodgkin lymphoma (NHL) patients treated at our centre. All patients were enrolled into a prospective longitudinal cohort study at a national teaching hospital and cancer referral centre serving half of Malawi's 16 million people. We included adult patients≥18 years of age with HIV-associated MCD (*n=*6), lymph node KS (*n=*5) or NHL (*n=*31) enrolled between 1 June 2013 and 31 January 2015.

**Results and discussion:**

MCD patients had a median age of 42.4 years (range 37.2–51.8). All had diffuse lymphadenopathy and five had hepatosplenomegaly. Concurrent KS was present for one MCD patient, and four had performance status ≥3. MCD patients had lower median haemoglobin (6.4 g/dL, range 3.6–9.3) than KS (11.0 g/dL, range 9.1–12.0, *p=*0.011) or NHL (11.2 g/dL, range 4.5–15.1, *p=*0.0007). Median serum albumin was also lower for MCD (2.1 g/dL, range 1.7–3.2) than KS (3.7 g/dL, range 3.2–3.9, *p=*0.013) or NHL (3.4 g/dL, range 1.8–4.8, *p=*0.003). All six MCD patients were on antiretroviral therapy (ART) with median CD4 count 208 cells/µL (range 108–1146), and all with HIV RNA <400 copies/mL. Most KS and NHL patients were also on ART, although ART duration was longer for MCD (56.4 months, range 18.2–105.3) than KS (14.2 months, range 6.8–21.9, *p=*0.039) or NHL (13.8 months, range 0.2–98.8, *p=*0.017). Survival was poorer for MCD patients than lymph node KS or NHL.

**Conclusions:**

HIV-associated MCD occurs in Malawi, is diagnosed late and is associated with high mortality. Improvements in awareness, diagnostic facilities, treatment and supportive care are needed to address this likely under-recognized public health problem in SSA.

## Introduction

HIV-associated multicentric Castleman disease (MCD) is a lymphoproliferative disorder caused by Kaposi sarcoma-associated herpesvirus (KSHV) [1–5]. Unlike Kaposi sarcoma (KS), MCD incidence may be increasing in the antiretroviral therapy (ART) era [6]. MCD is characterized by waxing and waning systemic inflammatory symptoms, lymphadenopathy and hepatosplenomegaly. The disease course may be indolent or fulminant, and survival among rituximab-treated patients in resource-rich settings currently exceeds 90% [1–5]. Laboratory abnormalities include anaemia, thrombocytopenia, hyponatremia, hypoalbuminaemia, and elevations of C-reactive protein (CRP), KSHV viral load, interleukin-6 (IL-6), and interleukin-10 (IL-10). Blood KSHV DNA and inflammatory markers correlate with disease activity. Patients can be critically ill, and often died within two years until recent therapies became available.

KS burden is high in sub-Saharan Africa (SSA), but clinical descriptions of MCD are infrequent despite high overlapping prevalence of HIV and KSHV. This may reflect underdiagnosis, as MCD has occurred among African immigrants in the United States [2]. In Uganda, a pathology review demonstrated latency-associated nuclear antigen (LANA) and viral IL-6 positivity by immunohistochemistry (IHC) in 2 of 64 reactive lymph nodes, suggesting MCD [7]. A pathology review from South Africa reported many HIV-associated B-cell lymphoproliferations without MCD or LANA staining described [8].

In Malawi, KS accounts for one-third of all cancers, yet we recently reported the first confirmed MCD case [9,10]. We have since identified several additional cases, and our objective in this paper is to describe clinical characteristics and survival of HIV-positive MCD patients in Lilongwe. We compare this group to patients with HIV-associated lymph node KS and non-Hodgkin lymphoma (NHL) simultaneously enrolled in an ongoing cohort study. Patients with clearly evident skin KS and lymphadenopathy are typically treated for KS without a biopsy in Malawi given severely limited pathology services, leading to under-represented lymph node KS in our cohort. Nevertheless, this is to our knowledge the first clinical MCD case series from SSA. Our experience suggests that MCD occurs with appreciable frequency and may be significantly under-recognized.

## Methods

Kamuzu Central Hospital (KCH) is the cancer referral centre for half of nearly 16 million people in Malawi, which is a low-income country in Southern Africa with 10–11% HIV prevalence, 83% ART coverage and annual gross domestic product per capita of US$355 [11,12]. We report data from the KCH Lymphoma Study, a prospective observational cohort initiated in June 2013. Patients with suspected or newly diagnosed lymphoproliferative disorders are eligible to participate. The main study objective is to prospectively characterize the spectrum of lymphoproliferative diseases in Malawi using enhanced pathology services, as well as treatment and outcomes under local conditions. All diagnoses are pathologically confirmed using tissue biopsies or cell blocks from centrifuged fine needle aspirates, with IHC and weekly real-time telepathology consultation involving two to four pathologists in Lilongwe and Chapel Hill who render a consensus opinion [13,14]. Given limited operating theatre availability and imaging-guided biopsy capacity, core or incisional biopsies are typically done at the bedside at the most clinically accessible site, with repeat biopsies done only when initial specimens are inconclusive. Available IHC stains include CD3, CD20, CD30, CD45, CD138, Ki-67, terminal deoxynucleotidyl transferase (TDT) and LANA. All MCD diagnoses are based on compatible clinical presentations and lymph node morphology by haematoxylin and eosin staining, as well as characteristic LANA IHC-positive lymphocytes, plasma cells and plasmablasts in the perifollicular and mantle zones as assessed during consensus telepathology review.

In an effort to use study resources to augment care locally and provide accurate diagnoses to patients and providers, while capturing all lymphoproliferative disorders at our centre, patients can be enrolled with confirmed lymphoproliferative disorders or clinical suspicion by referring providers. To enhance care locally, we have not attempted to strictly define criteria for study referral beyond a need for pathologic confirmation of clinically suspected lymphoma, absence of a prior confirmed lymphoproliferative condition and willingness to provide informed consent. Patients receive a comprehensive baseline evaluation and are followed for five years with active tracing and transportation reimbursement to promote retention. Staging for all patients includes chest x-ray and abdominal ultrasound, as well as unilateral bone marrow examination for NHL patients. Positron emission tomography is not available and computed tomography is not reliably available in the public sector. In Malawi, CD4 count monitoring is not done routinely, and ART is typically initiated based on World Health Organization clinical staging. Together with poor medical record systems, this makes it difficult to provide detailed descriptions of HIV clinical course including nadir CD4 cell count prior to study enrolment, other than data obtained at the time of enrolment. The University of North Carolina Institutional Review Board and Malawi National Health Sciences Research Committee approved this study.

For these analyses, we included adult patients ≥18 years of age with HIV-associated MCD, lymph node KS or NHL enrolled between 1 June 2013 and 31 January 2015. Differences between MCD, KS and NHL were assessed using Fisher's exact tests, one-way analysis of variance (ANOVA) and Kruskal–Wallis tests. Follow-up time was calculated from cohort enrolment until censoring, death or loss to follow-up, with censoring on 31 January 2015. Kaplan–Meier curves were used to estimate overall survival, and the log-rank test to assess survival differences between groups. All analyses were conducted using Stata version 12.1 (College Station, TX).

## Results

**Figure 1:**
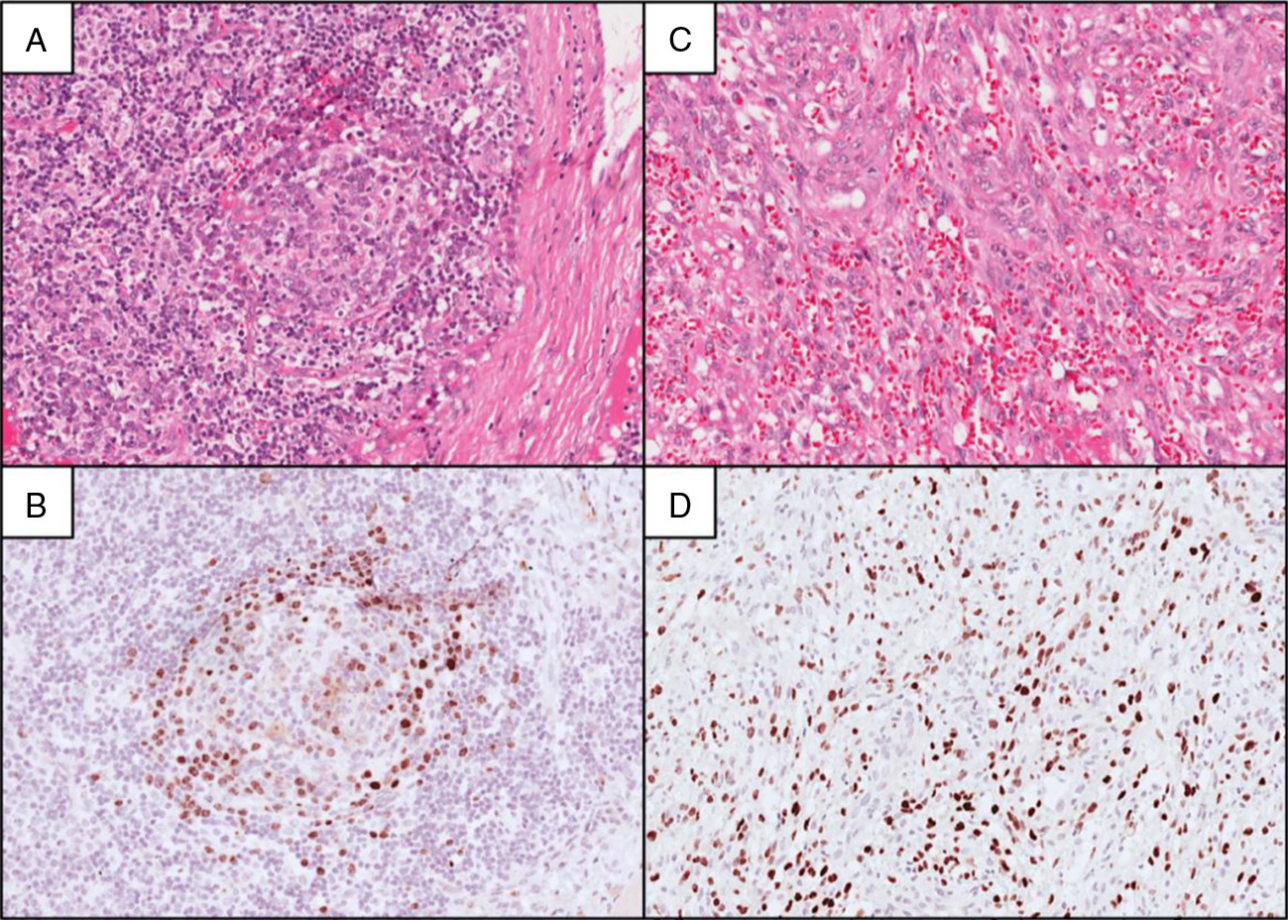
Multicentric Castleman disease and lymph node Kaposi sarcoma in Malawi. A lymph node section stained with haematoxylin and eosin in panel A shows an involuted and partially hyalinized germinal centre, as well as increased plasma cells in interfollicular regions. Corresponding latency-associated nuclear antigen (LANA) immunohistochemical staining in panel B demonstrates numerous LANA-positive lymphoid cells including larger plasmablasts localized to the perifollicular and mantle zones. In panel C, a lymph node section is shown with normal nodal architecture effaced by Kaposi sarcoma, characterized by spindle-shaped malignant epithelial cells lining slit-like vascular spaces. The spindle-shaped malignant cells are LANA-positive as shown in panel D.

During the study period, 95 adults ≥18 years of age were enrolled. Of these, 56 (59%) were HIV-positive, with pathologic diagnoses as follows: thirty-two NHL, six tuberculosis, six MCD, five KS without MCD, three benign/reactive, two Hodgkin lymphoma, one hepatocellular carcinoma and one non-diagnostic. All lymph nodes diagnosed as reactive were LANA IHC-negative. All HIV-associated MCD cases were further classified as the plasma cell histologic variant. Neither the hyaline vascular nor plasma cell variant of MCD was diagnosed in any HIV-negative patient. For these analyses, we included 42 patients with HIV-associated MCD (*n=*6), KS (*n=*5) or aggressive B-cell NHL (*n=*31). One HIV-positive NHL patient with Rai stage I chronic lymphocytic leukaemia not requiring therapy was excluded. During the study period, a seventh MCD case was pathologically confirmed in a lymph node specimen from a referring hospital, but we could not contact the patient or referring provider despite multiple attempts to engage the patient in care. This patient was therefore not enrolled or evaluated clinically at KCH and is not included. Representative biopsies from patients with MCD and lymph node KS are shown in Figure 1.

Clinical characteristics for MCD, KS and NHL patients are shown in Table 1. MCD patients had a median age of 42.4 years (range 37.2–51.8). Four were male, two reported symptom durations more than six months and four reported fever. All had diffuse lymphadenopathy with median largest lymph node 3 cm (range 2–4), and five had hepatosplenomegaly. Concurrent KS was present for one MCD patient, which was clinically evident on skin examination and pathologically evident in the lymph node specimen adjacent to areas involved by MCD. MCD patients were very ill with four having performance status ≥3. Impaired performance status was presumed secondary to MCD, as all patients had suppressed HIV RNA after receiving at least 18 months of ART, with no other evident HIV-related complications. In the sole patient with MCD and KS, systemic illness was markedly out of proportion to a limited burden of skin KS confined to the left medial thigh. Two patients were receiving empiric antituberculous treatment for their illness at the time of MCD diagnosis. Four KS patients without MCD had disease confined to lymph nodes (AIDS Clinical Trials Group T0). Of NHL patients, 20 (65%) had stage III/IV disease.

**Table 1:** Clinical characteristics of patients with HIV-associated multicentric Castleman disease, lymph node Kaposi sarcoma and aggressive B-cell non-Hodgkin lymphoma in Lilongwe, Malawi

	MCD (*n=*6)	Lymph node KS (*n*=5)	NHL (*n=*31)	*p* (MCD vs KS)	*p* (MCD vs NHL)
Age (years), median (range)	42.4 (37.2–51.8)	30.7 (25.6–33.8)	47.0 (22.4–62.6)	0.006	0.68
Male, *n* (%)	4 (66.7%)	2 (40.0%)	20 (64.5%)	0.57	1.00
Symptoms ≥6 months, *n* (%)	2 (33.3%)	1 (20.0%)	2 (6.4%)	1.00	0.12
Subjective fever, *n* (%)	4 (66.7%)	4 (80.0%)	11 (35.5%)	1.00	0.20
Receiving empiric antituberculous treatment, *n* (%)	2 (33.3%)	1 (20.0%)	3 (9.7%)	1.00	0.18
Localized adenopathy (vs diffuse), *n* (%)	0 (0.0%)	0 (0.0%)	13 (41.9%)	–	0.072
Largest lymph node mass (cm), median (range)	3 (2–4)	5 (2–7)	9 (2–30)	0.21	0.0009
Hepatosplenomegaly, *n* (%)	5 (83.3%)	0 (0.0%)	10 (32.3%)	0.015	0.031
Performance status ≥3, *n* (%)	4 (66.7%)	1 (20.0%)	6 (19.4%)	0.24	0.035
Mucocutaneous KS, *n* (%)	1 (16.7%)	1 (20.0%)	0 (0.0%)	1.00	0.16
White blood cells (10^3^/µL), median (range)	5.0 (2.3–17.1)	4.8 (3.7–8.5)	5.7 (3.0–12.0)	0.71	0.88
Haemoglobin (g/dL), median (range)	6.4 (3.6–9.3)	11.0 (9.1–12.0)	11.2 (4.5–15.1)	0.011	0.0007
Platelets (10^3^/µL), median (range)	84 (27–242)	180 (131–324)	222 (77–791)	0.14	0.012
Albumin (g/dL), median (range)	2.1 (1.7–3.2)	3.7 (3.2–3.9)	3.4 (1.8–4.8)	0.013	0.003
Lactate dehydrogenase (IU/L), median (range)^a^	294 (270–385)	185 (167–354)	479 (222–5570)	0.068	0.070
Antiretroviral therapy at enrolment, *n* (%)	6 (100.0%)	3 (60.0%)	28 (90.3%)	0.18	1.00
Duration (months), median (range)	56.4 (18.2–105.3)	14.2 (6.8–21.9)	13.8 (0.2–98.8)	0.039	0.017
CD4 (cells/µL), median (range)	208 (108–1146)	346 (49–607)	144 (24–2235)	0.86	0.15
HIV RNA (log_10_ copies/mL), median (range)	1.3 (1.3–2.6)	1.3 (1.3–5.5)	3.1 (1.3–7.0)	0.53	0.067
HIV RNA <400 copies/mL, *n* (%)	6 (100.0%)	3 (60.0%)	14 (45.2%)	0.18	0.022

MCD=multicentric Castleman disease; KS=Kaposi sarcoma; NHL=non-Hodgkin lymphoma;

a laboratory upper limit of normal is 250 IU/L.

Baseline laboratory assessments were performed at the time of pathologic confirmation. MCD patients had lower median haemoglobin (6.4 g/dL, range 3.6–9.3) than KS (11.0 g/dL, range 9.1–12.0, *p=*0.011) or NHL (11.2 g/dL, range 4.5–15.1, *p=*0.0007), and lower median platelet counts than NHL (84 versus 222×10^3^/µL, *p=*0.012). Median serum albumin was also lower for MCD (2.1 g/dL, range 1.7–3.2) than KS (3.7 g/dL, range 3.2–3.9, *p=*0.013) or NHL (3.4 g/dL, range 1.8–4.8, *p=*0.0031). Median lactate dehydrogenase for MCD patients tended to be higher than KS and lower than NHL. All six MCD patients were on ART at the time of MCD diagnosis with median CD4 count 208 cells/µL (range 108–1146), and all had suppressed HIV RNA <400 copies/mL. Most KS (60%) and NHL (90%) patients were also on ART at the time of KS or NHL diagnosis, although ART duration was longer for MCD cases (56.4 months, range 18.2–105.3) than KS (14.2 months, range 6.8–21.9, *p=*0.039) or NHL (13.8 months, range 0.2–98.8, *p=*0.017).

**Figure 2:**
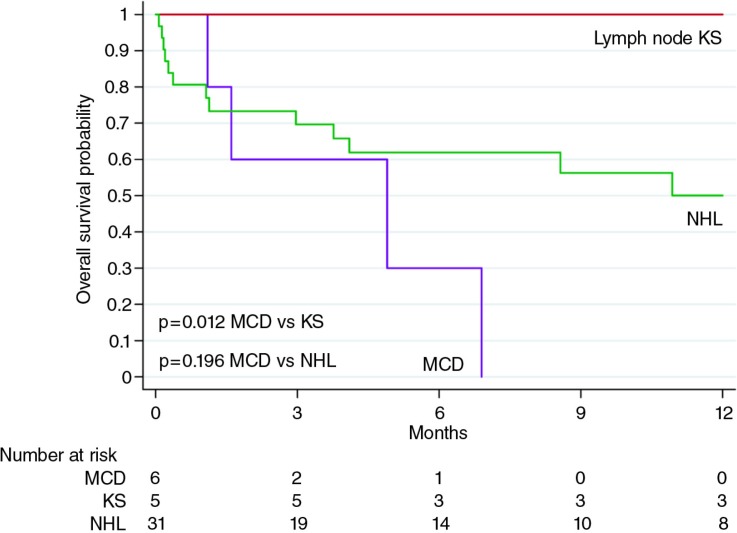
Overall survival for HIV-associated multicentric Castleman disease, lymph node Kaposi sarcoma and non-Hodgkin lymphoma at Kamuzu Central Hospital. KS=Kaposi sarcoma; NHL=non-Hodgkin lymphoma; MCD=multicentric Castleman disease.

Two MCD patients died before chemotherapy could be initiated. One of these was diagnosed with reactive lymphadenopathy just before LANA IHC was implemented in our laboratory, but was subsequently diagnosed with MCD after KSHV staining. The patient was contacted to return but died in the interim at home. The second patient was too ill for placement of intravenous access, and died while attempts were being made to acquire oral etoposide. Another MCD patient died two days after receiving a first dose of single-agent etoposide. Yet another MCD patient responded well to four doses of weekly etoposide, and returned to work after resolution of physical examination and laboratory abnormalities. However, death occurred four weeks after the last etoposide dose due to relapsed MCD before additional chemotherapy could be initiated. Currently, two MCD patients remain in active follow-up, one of whom has responded well to etoposide and another who is responding well to cyclophosphamide, vincristine and prednisone after failing to respond to two doses of etoposide, although treatment has been complicated by microbiologically confirmed pulmonary tuberculosis developing after chemotherapy initiation. By comparison, HIV-associated NHL patients in Malawi receive cyclophosphamide, doxorubicin, vincristine and prednisone, and of five lymph node KS patients, two received ART alone and three received ART plus chemotherapy (2 bleomycin, vincristine; 1 doxorubicin, bleomycin and vincristine). As of 31 January 2015, no patients were lost to follow-up. Kaplan–Meier overall survival is shown in Figure 2, suggesting worse survival for MCD compared to lymph node KS and NHL. Autopsies were not performed, although MCD was the presumed cause of death for all MCD patients based on available clinical information.

## Discussion

To our knowledge, this is the first clinical case series of HIV-associated MCD from SSA, a region with high HIV and KSHV prevalence. Our report suggests that MCD occurs with appreciable frequency in Malawi and is associated with high mortality. MCD patients in Lilongwe are very ill with worse performance status, anaemia and hypoalbuminaemia than have been described for contemporary HIV-associated MCD cohorts in resource-rich countries [5,15,16]. We agree with others that reported low MCD frequency in SSA reflects underdiagnosis and are unaware that a convincing biologic explanation exists for this observation otherwise. Greater MCD awareness is needed among clinicians and pathologists in SSA. The typical clinical presentation among our patients includes diffuse non-bulky lymphadenopathy with fever and hepatosplenomegaly, marked anaemia and hypoalbuminaemia, especially after long-term HIV control. Concurrent KS was not present in most of our MCD patients.

Limited pathology services contribute to underdiagnosis of MCD in SSA [13,14,17,18]. MCD histology can overlap with features of HIV infection, and equivocal cases are difficult to diagnose without KSHV stains. KS is often diagnosed clinically in Malawi and concurrent MCD may be missed. Missed concurrent MCD may partially explain more frequently detected plasma KSHV DNA and higher mortality for KS patients in SSA compared to resource-rich countries, as well as associations between anaemia and mortality for KS patients in Malawi and Uganda [19–21]. Greater awareness and facilities for MCD diagnosis and treatment may become even more important as ART scale-up continues. In Malawi, more than 80% of those needing ART receive it [11]. Our MCD patients were on ART for a median of nearly five years all with suppressed HIV RNA, suggesting disease burden in HIV-positive populations in SSA will undergo important epidemiologic shifts as ART availability increases, just as has occurred in resource-rich settings.

Overall survival among MCD patients in our cohort was very poor, stemming from late diagnosis, inadequate supportive care and inadequate disease-directed therapies. Even if better MCD treatments are available, weak acute care facilities in SSA may be a persistent obstacle, given that intensive supportive care is often needed to manage severe systemic inflammation in MCD. Although responses to chemotherapy are transient in MCD, we have treated patients with chemotherapy due to public sector availability in Malawi and lack of alternatives, while attempting to balance chemotherapy risks and benefits for a frail population in an environment with major supportive care limitations [22]. Disease-directed MCD treatments, including rituximab and virus-activated cytotoxic therapy with zidovudine and ganciclovir, are neither available nor well studied in Malawi. Anti-IL-6 agents are neither approved nor well-studied for KSHV-associated MCD even in resource-rich settings. We have recently advocated for a dedicated study of rituximab in NHL patients from SSA to demonstrate safety, efficacy and feasibility in settings like ours, given impending patent expiration, successful use of an existing biosimilar and increasing experience with subcutaneous administration which enhances feasibility for SSA [23–25]. These arguments are equally apt for MCD. Regardless, our experience and poor outcomes suggest a better approach is needed for MCD in resource-limited settings in SSA, and collaboration across centres will be important to define optimal management.

Strengths of our study include prospective, longitudinal follow-up of pathologically confirmed MCD, KS and NHL cases in a mature national ART programme, with no patients lost to date. Additionally, the ongoing KCH Lymphoma Study provides a unique platform with augmented pathology and clinical resources to support MCD confirmation, which would otherwise be absent in Lilongwe, in a region where this disease has largely been clinically unrecognized to date.

Study limitations include few MCD cases, and referral bias at a national teaching hospital. The Malawi health system suffers from extreme scarcity of health services particularly in rural areas, requiring patients to often travel great distances for higher-level care including diagnostic pathology and chemotherapy. This precludes estimates of population-level incidence, and may lead to partial characterization of MCD clinical spectrum, as more severe cases may die before presenting to KCH and less severe cases may not present at all. Our KS comparison group was enrolled in the same cohort study and underwent identical procedures, but predominantly had lymph node KS only and do not reflect the overall Lilongwe KS population. Furthermore, ancillary studies that are often incorporated into MCD diagnostic criteria (including KSHV viral load, CRP, IL-6, viral IL-6 IHC) are not available in Malawi, illustrating a need for diagnostic criteria applicable to SSA. If the full spectrum of KSHV-associated diseases is to be better understood, improved laboratory capacity will be required to support diagnosis of MCD and the related KSHV inflammatory cytokine syndrome in SSA [1–5,16–26].

## Conclusions

HIV-associated MCD occurs in Malawi and presents significant diagnostic and treatment challenges. Confirmed patients at our centre have been very ill with high mortality when managed with available resources. Addressing MCD in Malawi requires improved awareness, diagnostic facilities, supportive care and disease-directed treatments to improve outcomes for this likely under-recognized public health problem.

## References

[1] Bower M (2010). How I treat HIV-associated multicentric Castleman disease. Blood.

[2] Uldrick TS, Polizzotto MN, Yarchoan R (2012). Recent advances in Kaposi sarcoma herpesvirus-associated multicentric Castleman disease. Curr Opin Oncol.

[3] Cesarman E (2014). Gammaherpesviruses and lymphoproliferative disorders. Annu Rev Pathol.

[4] Polizzotto MN, Uldrick TS, Hu D, Yarchoan R (2012). Clinical manifestations of Kaposi Sarcoma herpesvirus lytic activation: multicentric Castleman disease (KSHV-MCD) and the KSHV inflammatory cytokine syndrome. Front Microbiol.

[5] Bower M, Newsom-Davis T, Naresh K, Merchant S, Lee B, Gazzard B (2011). Clinical features and outcome in HIV-associated multicentric Castleman's disease. J Clin Oncol.

[6] Powles T, Stebbing J, Bazeos A, Hatzimichael E, Mandalia S, Nelson M (2009). The role of immune suppression and HHV-8 in the increasing incidence of HIV-associated multicentric Castleman's disease. Ann Oncol.

[7] Engels EA, Mbulaiteye SM, Othieno E, Gomez M, Mathew S, Cesarman E (2007). Kaposi sarcoma-associated herpesvirus in non-Hodgkin lymphoma and reactive lymphadenopathy in Uganda. Hum Pathol.

[8] Wiggill TM, Mantina H, Willem P, Perner Y, Stevens WS (2011). Changing pattern of lymphoma subgroups at a tertiary academic complex in a high-prevalence HIV setting: a South African perspective. J Acquir Immune Defic Syndr.

[9] Msyamboza KP, Dzamalala C, Mdokwe C, Kamiza S, Lemerani M, Dzowela T (2012). Burden of cancer in Malawi; common types, incidence and trends: national population-based cancer registry. BMC Res Notes.

[10] Gopal S, Fedoriw Y, Montgomery ND, Kampani C, Krysiak R, Sanders MK (2014). Multicentric Castleman's disease in Malawi. Lancet.

[11] UNAIDS Malawi progress report for 2013 [Internet].

[12] United Nations Statistics Division Malawi country profile [Internet].

[13] Gopal S, Krysiak R, Liomba G (2013). Building a pathology laboratory in Malawi. Lancet Oncol.

[14] Gopal S, Krysiak R, Liomba NG, Horner MJ, Shores CG, Alide N (2013). Early experience after developing a pathology laboratory in Malawi, with emphasis on cancer diagnoses. PLoS One.

[15] Uldrick TS, Polizzotto MN, Aleman K, O'Mahony D, Wyvill KM, Wang V (2011). High-dose zidovudine plus valganciclovir for Kaposi sarcoma herpesvirus-associated multicentric Castleman disease: a pilot study of virus-activated cytotoxic therapy. Blood.

[16] Gerard L, Berezne A, Galicier L, Meignin V, Obadia M, De Castro N (2007). Prospective study of rituximab in chemotherapy-dependent human immunodeficiency virus associated multicentric Castleman's disease: ANRS 117 CastlemaB Trial. J Clin Oncol.

[17] Adesina A, Chumba D, Nelson AM, Orem J, Roberts DJ, Wabinga H (2013). Improvement of pathology in sub-Saharan Africa. Lancet Oncol.

[18] Naresh KN, Raphael M, Ayers L, Hurwitz N, Calbi V, Rogena E (2011). Lymphomas in sub-Saharan Africa – what can we learn and how can we help in improving diagnosis, managing patients and fostering translational research?. Br J Haematol.

[19] Letang E, Lewis JJ, Bower M, Mosam A, Borok M, Campbell TB (2013). Immune reconstitution inflammatory syndrome associated with Kaposi sarcoma: higher incidence and mortality in Africa than in the UK. AIDS.

[20] Laker-Oletta MO, Glidden DV, Walusansa V, Orem J, Mocello AR, Maurer T (2015). Prognostic model for patients with Kaposi Sarcoma treated with ART alone in Africa [abstract 88].

[21] Herce ME, Kalanga N, Wroe EB, Keck JW, Chingoli F, Tengatenga L (2015). Excellent clinical outcomes and retention in care for adults with HIV-associated Kaposi sarcoma treated with systemic chemotherapy and integrated antiretroviral therapy in rural Malawi. J Int AIDS Soc.

[22] Gopal S, Wood WA, Lee SJ, Shea TC, Naresh KN, Kazembe PN (2012). Meeting the challenge of hematologic malignancies in sub-Saharan Africa. Blood.

[23] Roy PS, John S, Karankal S, Kannan S, Pawaskar P, Gawande J (2013). Comparison of the efficacy and safety of Rituximab (Mabthera) and its biosimilar (Reditux) in diffuse large B-cell lymphoma patients treated with chemo-immunotherapy: a retrospective analysis. Indian J Med Paediatr Oncol.

[24] Salar A, Avivi I, Bittner B, Bouabdallah R, Brewster M, Catalani O (2014). Comparison of subcutaneous versus intravenous administration of rituximab as maintenance treatment for follicular lymphoma: results from a two-stage, phase IB study. J Clin Oncol.

[25] Davies A, Merli F, Mihaljevic B, Siritanaratkul N, Solal-Celigny P, Barrett M (2014). Pharmacokinetics and safety of subcutaneous rituximab in follicular lymphoma (SABRINA): stage 1 analysis of a randomised phase 3 study. Lancet Oncol.

[26] Bower M, Pria AD, Coyle C, Nelson M, Naresh K (2014). Diagnostic criteria schemes for multicentric Castleman disease in 75 cases. J Acquir Immune Defic Syndr.

